# Environmental health and women’s health nursing

**DOI:** 10.4069/whn.2024.08.20

**Published:** 2024-09-30

**Authors:** Ju Hee Kim

**Affiliations:** College of Nursing Science, Kyung Hee University, Seoul, Korea

## Introduction

The environment is a major metaparadigm of nursing, and environmental health is an important topic in women’s health nursing. Environmental health focuses on the interaction between human health and the environment, and women’s health nursing deals with health and nursing care throughout a woman’s life cycle. In particular, women face various health challenges due to biological, social, and cultural factors; therefore, it is important to understand how women are affected by various environmental factors. Understanding these impacts will enable us to provide appropriate care for women. In this editorial, I describe the effects of environmental hazards on women’s health.

## Environmental hazards and women’s health

Environmental hazards primarily consist of air pollutants and chemical substances. The latter category encompasses endocrine-disrupting chemicals such as phthalates, bisphenols, and parabens, as well as persistent organic pollutants like perfluorinated compounds and dioxins, and heavy metals including lead, cadmium, mercury, and arsenic. Air pollutants comprise fine dust particles (PM_10_, PM_2.5_, PM_1.0_), ozone (O_3_), nitrogen dioxide (NO_2_), carbon monoxide (CO), sulfur dioxide (SO_2_), and volatile organic compounds (VOCs), all of which are inhaled from the air. These substances can enter the human body through food consumption, inhalation, or skin contact. Following these exposure pathways, these pollutants have been detected in various human samples, such as urine, blood, saliva, hair, breast milk, and placenta [1-3].

Environmental diseases, including respiratory and skin conditions, infectious diseases, and cancer, are on the rise due to a variety of complex factors such as climate change, air pollution, increased plastic use, and water pollution. Exposure to these environmental hazards poses significant risks to environmentally sensitive or vulnerable groups, including women of reproductive age, pregnant women, mothers, and infants. Such exposure can increase the likelihood of developing conditions like polycystic ovary syndrome, endometriosis, and uterine fibroids. It can also lead to miscarriage, premature birth, gestational diabetes, high blood pressure during pregnancy, infertility, and postpartum depression, and even affect the neurological development of infants and toddlers [4-9].

## Women’s biological vulnerability

Women are an environmentally sensitive group and may be more vulnerable to certain environmental hazards than men due to biological and physiological factors. For example, hormonal fluctuations involving estrogen and progesterone can heighten women’s sensitivity to certain chemicals. Progesterone, specifically, reduces gastrointestinal motility and relaxes smooth muscles, thereby prolonging the retention of chemicals in the gastrointestinal tract by 30% to 50% [7]. This increased retention time heightens susceptibility to harmful agents. Moreover, pregnant women face additional risks as they are exposed to fine particulate matter and various air pollutants such as VOCs, SO_2_, NO_2_, and O_3_ through both indoor and outdoor activities. They also encounter endocrine disruptors like phthalates and bisphenols in everyday products. During pregnancy, the respiratory rate and cardiac output of a woman increase by approximately 30% to 40% compared to pre-pregnancy levels, potentially leading to greater exposure to these harmful substances [8]. The environmental hazards absorbed by pregnant women can be transferred to the fetus via the placenta or to the newborn through breastfeeding. Most environmental hazards have a molecular weight under 600 Da, facilitating their passage through the placenta.

## Role of nursing

The role of nurses in environmental health is very important. Nurses and nursing researchers must study the effects of various environmental hazards on women’s health and develop prevention and management strategies across the women’s life cycle. Based on these studies, preventive nursing care should be provided to the subjects. For instance, nurses can inform women about how to reduce their exposure to chemicals and air pollutants. A recent study reported that nurses provided 10 lifestyle modifications to reduce exposure to endocrine disruptors over a 4-week period to infants and their mothers, resulting in a 55.7% decrease in urinary bisphenol and paraben levels [5]. Furthermore, nurses can provide more than just information; they can also assist patients in increasing their environmental health literacy (EHL), which includes strategies to avoid exposure and select safe products. In a recent randomized controlled trial, nurses conducted a 1-week intervention for women of reproductive age. This intervention not only reduced their exposure to fine dust and air pollutants but also enhanced their EHL [10].

## Policy approach

Policy approaches are essential for protecting women’s health. Governments and health authorities must strengthen environmental health management and standards and develop policies to protect women’s health. For example, in 2017, the Korea Ministry of Food and Drug Safety conducted an investigation into the hazardous chemicals present in sanitary napkins and verified that the concentration of VOCs in most products was below the established safety standards. There is a pressing need to strengthen the safety monitoring of products and to set stringent legal standards that rigorously control the use of chemicals. In this context, the role of nurses specializing in women’s health becomes significantly important. Moreover, ongoing research into the link between women’s health and environmental hazards is essential. Developing health policies based on research findings will enable us to support women in leading healthier lives within safer environments.

## Conclusion

Environmental health and women’s health nursing are closely connected, and a multifaceted approach is needed to help women, who constitute an environmentally sensitive/vulnerable group. This requires a combination of individual, social, and policy-level interventions. Nurses are pivotal in these efforts, playing a crucial role in safeguarding and enhancing women’s health. The overarching aim is to protect women from environmental hazards and enable them to lead healthy lives, supported by ongoing research, education, and policy initiatives.
